# Clinically stable covid-19 patients presenting to acute unscheduled episodic care venues have increased risk of hospitalization: secondary analysis of a randomized control trial

**DOI:** 10.1186/s12879-023-08295-9

**Published:** 2023-05-15

**Authors:** Joseph Bledsoe, Scott C. Woller, Maria Brooks, Frank C. Sciurba, Jerry A. Krishnan, Deborah Martin, Peter Hou, Janet Y. Lin, Andrei Kindzelski, Eileen Handberg, Bridget-Anne Kirwan, Elaine Zaharris, Lauren Castro, Nancy L. Shapiro, Carl J. Pepine, Sarah Majercik, Zhuxuan Fu, Yongqi Zhong, Vidya Venugopal, Yu-Hsuan Lai, Paul M. Ridker, Jean M. Connors

**Affiliations:** 1 Intermountain Healthcare, Emergency Medicine and Trauma, Salt Lake City, UT USA; 2 Intermountain Medical Center, Department of Medicine, 5121 S. Cottonwood Drive, Murray, UT 84157 USA; 3 School of Medicine, University of Pittsburgh, Pittsburgh, PA USA; 4 Division of Pulmonary, Critical Care, Sleep, and Allergy, University of Illinois, Chicago, IL USA; 5 Brigham and Women’s Hospital, Boston, MA USA; 6 Department of Emergency Medicine, University of Illinois, Chicago, IL USA; 7 National Heart, Lung, and Blood Institute, Bethesda, MD USA; 8 School of Medicine, University of Florida, Gainesville, FL USA; 9 SOCAR Research SA, Nyon, Switzerland; 10 Department of Pharmacy Practice, College of Pharmacy, University of Illinois, Chicago, IL USA

**Keywords:** SARS-CoV-2, COVID-19, Pulmonary embolism, PE, Venous thromboembolic disease, VTE, Stroke, CVA, Clinical trial enrollment

## Abstract

**Background:**

Assessment for risks associated with acute stable COVID-19 is important to optimize clinical trial enrollment and target patients for scarce therapeutics. To assess whether healthcare system engagement location is an independent predictor of outcomes we performed a secondary analysis of the ACTIV-4B Outpatient Thrombosis Prevention trial.

**Methods:**

A secondary analysis of the ACTIV-4B trial that was conducted at 52 US sites between September 2020 and August 2021. Participants were enrolled through acute unscheduled episodic care (AUEC) enrollment location (emergency department, or urgent care clinic visit) compared to minimal contact (MC) enrollment (electronic contact from test center lists of positive patients).We report the primary composite outcome of cardiopulmonary hospitalizations, symptomatic venous thromboembolism, myocardial infarction, stroke, transient ischemic attack, systemic arterial thromboembolism, or death among stable outpatients stratified by enrollment setting, AUEC versus MC. A propensity score for AUEC enrollment was created, and Cox proportional hazards regression with inverse probability weighting (IPW) was used to compare the primary outcome by enrollment location.

**Results:**

Among the 657 ACTIV-4B patients randomized, 533 (81.1%) with known enrollment setting data were included in this analysis, 227 from AUEC settings and 306 from MC settings. In a multivariate logistic regression model, time from COVID test, age, Black race, Hispanic ethnicity, and body mass index were associated with AUEC enrollment. Irrespective of trial treatment allocation, patients enrolled at an AUEC setting were 10-times more likely to suffer from the adjudicated primary outcome, 7.9% vs. 0.7%; p < 0.001, compared with patients enrolled at a MC setting. Upon Cox regression analysis adjustment patients enrolled at an AUEC setting remained at significant risk of the primary composite outcome, HR 3.40 (95% CI 1.46, 7.94).

**Conclusions:**

Patients with clinically stable COVID-19 presenting to an AUEC enrollment setting represent a population at increased risk of arterial and venous thrombosis complications, hospitalization for cardiopulmonary events, or death, when adjusted for other risk factors, compared with patients enrolled at a MC setting. Future outpatient therapeutic trials and clinical therapeutic delivery programs of clinically stable COVID-19 patients may focus on inclusion of higher-risk patient populations from AUEC engagement locations.

**Trial Registration:**

ClinicalTrials.gov Identifier: NCT04498273.

## Introduction

The SARS-CoV-2 virus has had significant global impact and COVID-19 has been linked to arterial and venous thromboses as well pulmonary vascular micro-thrombosis on autopsy [1–11]. Patients with COVID-19 often experience thrombotic complications early in the course of hospitalization suggesting their presence on admission [12]. As such, anticoagulant and antiplatelet therapies initiated prior to hospitalization was hypothesized to reduce both micro- and macrovascular -thrombotic complications [13].

The ACTIV-4B Outpatient Thrombosis Prevention trial was a randomized, double-blind placebo-controlled trial comparing aspirin 81 mg once daily, apixaban 2.5 mg twice daily, apixaban 5.0 mg twice daily among symptomatic patients with COVID-19 not initially requiring hospitalization. The trial primary endpoint was a composite outcome of all-cause mortality, symptomatic venous or arterial thromboembolism, myocardial infarction, stroke, or hospitalization for cardiopulmonary cause [14]. The trial concluded early due to a lower than anticipated event rate without evidence of efficacy for either aspirin or apixaban as compared to placebo.

To optimize enrollment and facilitate study completion during a global pandemic, the ACTIV-4B trial adopted a trial design that permitted remote patient enrollment using electronic consent with contact initiated by the study team from electronic health record (EHR) generated lists of patients testing positive for COVID-19 (minimal contact—MC) as an alternative to traditional in-person enrollment at acute unscheduled episode care (AUEC) settings, such as walk-in COVID-19 clinics, urgent care clinics and emergency departments (ED). In this secondary analysis of the ACTIV-4B clinical trial we evaluate the effect of enrollment setting, MC versus AUEC, on the rates of the trial primary composite endpoint. Additionally, we describe phenotypical differences observed in patients based on enrollment location.

**Table 1:** Patient baseline characteristics

Characteristic	All Randomized (N = 533)	Minimal Contact enrollment (N = 306)	Acute Episodic Care enrollment (N = 227)	p-value^#^
Enrollment Location—no. (%)				
Emergency department visit	95 (17.8)	-	95 (41.9)	
Urgent care visit	23 (4.3)	-	23 (10.1)	
Monoclonal antibody or COVID clinic visit	88 (16.5)	-	88 (38.8)	
Other urgent clinic visits	21 (3.9)	-	21 (9.3)	
Low/No- Touch enrollment	306 (57.4)	306 (100.0)	-	
Median time from COVID test to randomization (IQR)—days	6.0 (3.0, 10.0)	9.0 (5.0, 12.0)	3.0 (1.0, 6.0)	< 0.001
Participants who initiated trial treatment–no.	458	280	178	
Median time from COVID test to treatment initiation (IQR)—days	11.0 (7.0, 14.0)	13.0 (10.0, 15.0)	8.0 (5.0, 11.0)	< 0.001
Median time from randomization to treatment initiation (IQR)—days	3.0 (2.0, 5.0)	3.0 (2.0, 5.0)	3.0 (2.0, 5.0)	0.62
Median age (IQR*)—yr	54.0 (46.0, 59.0)	52.0 (45.0, 58.0)	55.0 (48.0, 60.0)	0.005
Sex—no. (%)				
Female	311 (58.4)	183 (59.8)	128 (56.4)	0.43
Male	222 (41.7)	123 (40.2)	99 (43.6)	
Race / ethnicity —no. (%)				
Black non-Hispanic	61 (11.4)	19 (6.2)	42 (18.9)	< 0.001
Hispanic	98 (18.4)	32 (10.5)	66 (29.1)	
White non-Hispanic	339 (63.6)	231 (75.5)	108 (47.6)	
Other**	35 (6.6)	24 (7.8)	11 (4.9)	
Region-no (%)				
Northeast	47 (8.8)	40 (13.1)	7 (3.1)	< 0.001
Midwest	77 (14.5)	37 (12.1)	40 (17.6)	
South	236 (44.3)	59 (19.3)	177 (78.0)	
West	173 (32.5)	170 (55.6)	3 (1.3)	
Median body mass index (IQR)—kg/m^2^	30.1 (26.0, 35.4)	29.3 (25.5, 33.9)	31.4 (27.3, 37.7)	< 0.001
History of DVT or PE – no. (%)	19 (3.6)	12 (3.9)	7 (3.1)	0.61
Hypertension—no. (%)	179 (33.6)	82 (26.8)	97 (42.7)	< 0.001
Diabetes—no. (%)	102 (19.1)	43 (14.1)	59 (26.0)	< 0.001
History of smoking—no. (%)	120 (22.5)	65 (21.2)	55 (24.2)	0.41
Median Platelet count (IQR)—per mm [3]	239.0 (189.0, 307.0)	274.0 (215.0, 329.0)	211.5 (169.0, 251.0)	< 0.001
Median creatinine clearance (IQR)—mg/ml/1.73m^2^	114.5 (91.3, 144.6)	113.0 (91.4, 144.7)	117.0 (91.0, 144.4)	0.5
D-dimer—no. (%)***	478	264	214	
≤1 X upper limit of normal	313 (65.5)	174 (65.9)	139 (65.0)	0.82
>1 - ≤2 X upper limit of normal	115 (24.1)	61 (23.1)	54 (25.2)	
>2 X upper limit of normal	50 (10.5)	29 (11.0)	21 (9.8)	
Median hsCRP (IQR)—mg/L	4.0 (1.5, 12.5)	3.0 (1.3, 10.0)	6.0 (1.9, 31.3)	< 0.001

*IQR denotes interquartile range. **Other includes American Indian, Alaska Native, Asian, Native Hawaiian or other Pacific Islander, and unknown. ***D-dimer assays varied from site to site. Upper limit of normal was captured for each site with individual participant results compared to local values to determine if within the normal range or elevated above the normal range.

*# p-values comparing characteristics in the Minimal Contact enrollment to those in the Acute Episodic Care enrollment group are based on Wilcoxon rank-sum statistics for continuous variables and chi-square statistics for categorical variables.*

## Methods

The ACTIV4B Outpatient Thrombosis Clinical trial was funded by Operation Warp Speed and conducted as part of the National Heart, Lung, and Blood Institute (NHLBI) ACTIV platform of clinical trials. The enrollment methods, primary trial protocol, patient population, enrollment centers and outcome measurement methods are previously described [14]. The protocol, informed consent documents and statistical analysis plan were approved by the Western Institutional Review Board (WIRB) and at each of the 52 centers in the US that participated in the trial. Patients enrolled in the trial provided written informed consent for participation and all research involving human participants, human material, or human data, was performed in accordance with the Declaration of Helsinki and was approved by an appropriate ethics committee.

**Table 2:** Unadjusted and multivariable adjusted odds ratios for acute episodic care enrollment versus Minimal Contact enrollment

N = 533	Unadjusted Odds Ratio (95% CI) for AEC versus MC enrollment	Multivariable Adjusted Odds Ratio (95% CI) for AEC versus MC enrollment
Time from COVID test to randomization, per day	0.77 (0.73, 0.81)	0.77 (0.73, 0.82)
Time from COVID-19 test to Randomization		--
0–2 days	1.0	
3–5 days	0.20 (0.11, 0.36)	
6–9 days	0.13 (0.07, 0.23)	
>=10 days	0.04 (0.02, 0.08)	
D-Dimer, per 1 unit	1.02 (0.88, 1.18)	--
D-Dimer		--
≤1.0 times ULN	1.0	
>1.0 - ≤2.0 times ULN	1.11 (0.72, 1.70)	
>2.0 times ULN	0.91 (0.50, 1.660	
Missing	0.39 (0.20, 0.75)	
Ln CRP, per 1 unit	1.23 (1.10, 1.38)	1.03 (0.90, 1.18)
CRP		--
≤2 mg/L	1.0	
> 2- ≤ 4 mg/L	0.94 (0.55, 1.59)	
> 4 mg/L	1.72 (1.15, 2.58)	
Missing	0.44 (0.16, 1.25)	
Ln Creatinine clearance, per 1 unit	1.15 (0.69, 1.90)	--
Age, per year	1.03 (1.01, 1.05)	1.04 (1.01, 1.07)
Age		--
40 - ≤50 years	1.0	
>50- ≤60 years	1.52 (1.03, 2.24)	
>60- ≤80 years	1.77 (1.11, 2.82)	
Sex		
Male	1.15 (0.81, 1.63)	1.17 (0.76, 1.81)
Female	1.0	1.0
Race/Ethnicity		
Black non-Hispanic	4.74 (2.64, 8.49)	4.38 (2.10, 9.10)
Hispanic	4.42 (2.75, 7.11)	5.94 (3.37, 10.47)
White non-Hispanic	1.0	1.0
Other Race/Ethnicity	1.0	1.0
Region		--
Northeast	1.0	
Midwest	6.18 (2.46, 15.49)	
South	17.14 (7.29, 40.32)	
West	0.10 (0.03, 0.41)	
Body mass index, per 1 kg/m^2^	1.06 (1.03, 1.09)	1.05 (1.02, 1.09)
History of Smoking		--
Yes	1.19 (0.79, 1.79)	
No/Missing	1.0	
Hypertension		
Yes	2.04 (1.42, 2.94)	1.29 (0.79, 2.10)
No/Missing	1.0	1.0
Diabetes		
Yes	2.15 (1.39, 3.33)	1.06 (0.60, 1.87)
No/Missing	1.0	1.0
History of DVT or PE		--
Yes	0.78 (0.30, 2.01)	
No/Missing	1.0	

For this secondary analysis we requested enrollment location data from the enrolling centers, information not originally collected as part of the ACTIV4B trial. Each site primary investigator and lead study coordinator completed a pre-populated table identifying each study participant as having been enrolled either at an AUEC setting, or through the MC process. AUEC enrolled patients included those COVID-19 positive patients (defined as having a positive PCR or antigen test within 14 days) seen and enrolled in an ED, urgent care, Monoclonal Antibody (MAB) clinic or acute care COVID-19 clinic for symptomatic COVID-19. Informed consent and enrollment occurred at or immediately following a visit at one of these settings. MC patients were those patients identified as COVID-19 positive via automated interrogation of the EHR that generated a COVID-19-positive test list, not otherwise meeting inclusion in the AUEC group. In the MC group each patient was contacted electronically via telephone, text message, or email, and the patient completed study orientation via an online video sent as a link in a text message or email. Inclusion/exclusion criteria were affirmed with a site investigator and signed consent was obtained. MC participants were also required to be COVID-19 test positive within 14 days of enrollment. Deidentified participant data from this trial will be made publicly available to researchers upon approval of use after 1 January 2023 by contacting the National Institutes of Health.

**Fig. 1:**
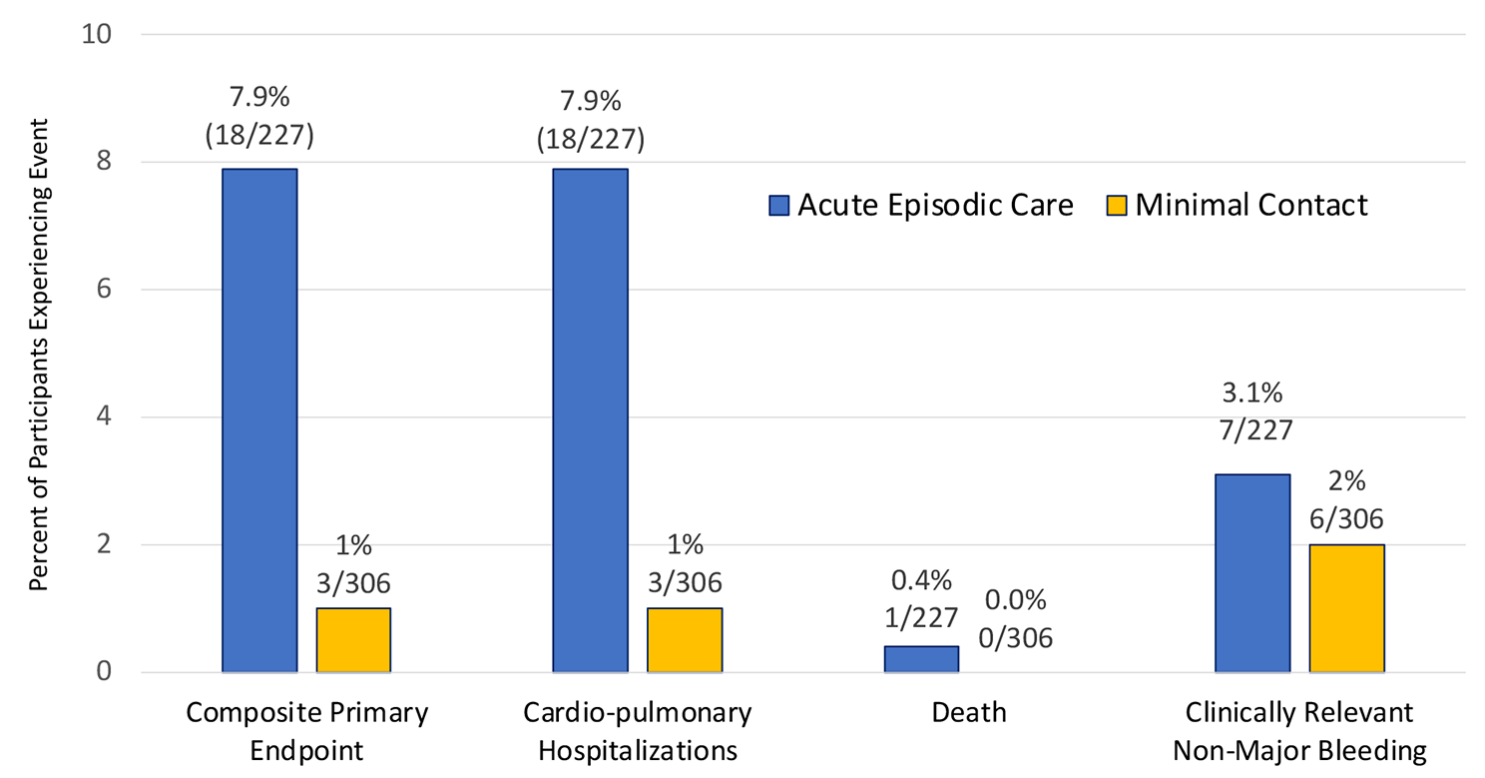
Risk of Key Endpoints for Acute Episodic Care and Minimal Contact Participants

### Statistical analysis

Baseline patient characteristics were compared across enrollment settings; frequencies (percentages) are presented for categorical variables and medians (first and third quartiles) for continuous variables. P-values comparing characteristics in the MC enrollment group to those in the AUEC enrollment group are based on Wilcoxon rank-sum statistics for continuous variables and chi-square statistics for categorical variables. A logistic regression model including sex and all variables, besides region, that were associated with AUEC were used to create a propensity score for AUEC enrollment. Odds ratios from the logistic regression models are reported with 95% confidence intervals.

**Table 3:** Primary Outcome and Bleeding Events

Characteristic	All Randomized (N = 533)	Minimal Contact enrollment (N = 306)	Acute Episodic Care enrollment (N = 227)	p-value^#^
Adjudicated primary outcomes—no. (%)				
Composite primary endpoint	20 (3.8)	2 (0.7)	18 (7.9)	< 0.001
Cardio-pulmonary hospitalizations	20 (3.8)	2 (0.7)	18 (7.9)	< 0.001
COVID-19 associated pneumonia	20 (3.8)	2 (0.7)	18 (7.9)	< 0.001
Deep vein thrombosis or pulmonary embolism	1 (0.2)	0 (0.0)	1 (0.4)	0.43
Myocardial infarction, stroke or other arterial embolism	0 (0.0)	0 (0.0)	0 (0.0)	-
Death	1 (0.2)	0 (0.0)	1 (0.4)	0.43
Suspected Hemorrhagic Events—no. (%)*				
Major bleeding	0 (0.0)	0 (0.0)	0 (0.0)	-
Clinically relevant non-major bleeding	13 (2.4)	6 (2.0)	7 (3.1)	0.41
Minor bleeding	17 (3.2)	8 (2.6)	9 (4.0)	0.46
**Adjudicated Hemorrhagic Events—no. (%)**				
Major bleeding	0 (0.0)	0 (0.0)	0 (0.0)	-
Clinically relevant non-major bleeding	5 (0.9)	2 (0.7)	3 (1.3)	0.66

*Suspected major and clinically relevant non-major bleeding events were reported by the trial medical monitor, and minor bleeding events were identified through follow-up with the research pharmacists. Major and clinically relevant non-major bleeding events were adjudicated by the Clinical Events Committee; minor bleeding events were not adjudicated.

*# p-values comparing outcome risk in the Minimal Contact enrollment to that in the Acute Episodic Care enrollment group are based on Fisher’s exact tests*.

The occurrence of the primary composite endpoint at trial completion in each enrollment location cohort was computed as a simple proportion. P-values comparing outcome risk in the minimal touch enrollment group to that in the AUEC enrollment group are based on Fisher’s exact tests. Since the number of trial outcome events was limited, the association between AUEC enrollment and the primary composite outcome was estimated using an unadjusted Cox regression model and an inverse probability weighted Cox regression model based on the propensity score for AUEC enrollment location. A multivariable inverse probability weighted Cox model adjusting for factors shown to predict the trial primary composite outcome (sex, race/ethnicity, time from COVID test and log of CRP level) was created as a sensitivity analysis. Estimated hazards ratios are reported with 95% confidence intervals. Analyses were conducted with SAS version 9.4 (SAS Institute Inc).

## Results

Patient population: From September 1, 2020, through June 17, 2021, 775 potential participants were screened and provided informed consent of whom 657 were randomized. The enrollment location was available for 533 (81.1%) of the 657 randomized patients, of which 306 (57%) were classified as MC enrollment patients and 227 (43%) were classified as AUEC enrollment patients. The median age was 52 years (interquartile range [IQR]: 45, 58 years) for the MC patients and 55 years (IQR: 48, 60 years; vs. MC, p-value = 0.005) for AUEC patients; overall 58.4% were female with no significant difference between groups (Table 1).

**Fig. 2:**
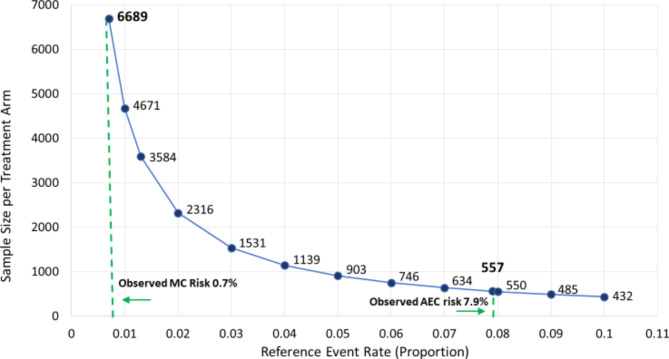
Sample Size required for each treatment arm to have 80% power to detect a 50% risk reduction with alpha = 0.05 for a range of reference event rates with estimates based on the adjudicated primary clinical outcome

When compared to MC enrollment patients, AUEC enrollment patients were enrolled sooner after symptom onset (median 3 days [IQR 1, 6 days] vs. 9 days [IQR 5, 12 days] days; p < 0.001) and initiated study treatment sooner (median 8 days (IQR 5, 11 days) vs. 13 days (IQR 10, 15 days), p < 0.001). AUEC enrollment patients were more likely to be non-Hispanic Black (18.9% vs. 6.2% p < 0.001) or Hispanic (29.1% vs. 10.5% p < 0.001) compared to MC enrollment patients. Regional enrollment differed such that AUEC patients were more often enrolled in the Southern US (78%) and MC patients were more often enrolled in Western US patients (55.6%, p < 0.001). In a multivariable- logistic regression model, significant predictors of AUEC enrollment as compared to MC enrollment included shorter time between positive COVID-test and randomization, OR = 0.77 per day (95% CI- 0.73, 0.82), older age, OR = 1.04 per year (95% CI 1.01, 1.07), Black non-Hispanic race/ethnicity, OR = 4.38 (95% CI 2.10, 9.10) Hispanic ethnicity, OR = 5.94 (95% CI 3.37, 10.47) and higher body mass index (BMI), OR = 1.05 per km/m^2^ (95% CI 1.02, 1.09) (Table 2). The multivariable logistic regression model shown in Table 2 was used to create the propensity score for AUEC enrollment.

Clinical Outcomes: Among the 533 patients with enrollment location data, 20 (3.8%) developed the adjudicated composite primary outcome, of which 18 of 227 (7.9%) occurred among the AUEC enrollment patients and two of 306 (0.7%) occurred among the MC enrollment patients (p < 0.001) (Fig. 1).

All 20 participants experiencing a primary composite endpoint were hospitalized for COVID-19 pneumonia and one also had VTE. One patient death occurred due to COVID-19 pneumonia and was in the AUEC enrollment group (Table 3). There were no significant differences for bleeding events between enrollment groups.

The unadjusted hazards of the adjudicated primary outcome were 12.57 (95% CI: 2.92, 54.19) higher for those enrolled through AUEC as compared to those enrolled through the MC pathway. In Cox regression analysis with inverse probability weighting using a propensity score accounting for age, sex, time from COVID test to randomization, race/ethnicity, log of CRP level, BMI, hypertension and diabetes, enrollment in the AUEC pathway remained significantly associated with the adjudicated primary outcome, adjusted HR 3.40 (95% CI 1.46, 7.93). The sensitivity analysis results from the multivariable-adjusted Cox model were consistent but attenuated, adjusted HR 2.46 (95% CI 1.03, 5.88).

## Discussion

This ACTIV-4B trial secondary analysis demonstrates that clinical trial enrollment setting is an important predictor of the composite outcome of hospitalization, venous or arterial thrombosis, stroke, myocardial infarction, or death in patients with clinical stable COVID-19. When adjusted for other risk factors the location of enrollment remains an important predictor of outcomes.

The underlying cause of this finding is likely multifactorial. One possible explanation is that patients that are more ill will self-select to an AUEC location out of concern for their health (as opposed to testing remotely) even though at first evaluation they were discharged for care at home. This hypothesis is supported by the observation that the median time to enrollment was shorter among AUEC patients compared with MC patie MC alternatives to AUEC may not have been uniformly available for all ill individuals. While broad awareness of AUECs in a community exists, MC assessment as a potential resource is comparatively novel in clinical research prior to the pandemic. Likewise, MC enrollment was incumbent on screening that occurred at sites that established this novel screening infrastructure. Some of these limitations may be more pronounced in certain areas such as neighborhoods with disproportionally more elderly, or among certain populations (e.g., Blacks and Hispanics). These findings may also highlight the important safety net provided by emergency departments and other AUEC settings in providing access to care or trial opportunities not otherwise readily available to vulnerable patients at elevated risk.

We identified phenotypical factors associated with COVID-19 clinical outcome events among patients presenting to an AUEC location or an MC location. These findings have ramifications that can inform future clinical trial design to optimize enrollment equity. While standardized enrollment criteria existed for ACTIV-4B, patients that presented to MC settings and AUEC settings differed. AUEC enrollment patients were more likely to be Black, Hispanic, and older. Some minimal contact COVID-19 clinical trials have had limited success in enrolling targeted numbers of patients [15]. Others have reported similar elevated risk for patients of various racial or ethnical backgrounds, in addition to older age and BMI [14, 16–19]. Strategies to enroll patients in clinical trials have focused on individual patient characteristics [20]; however our study demonstrates that the location in which patients seek care has implications for risk of experiencing an event even when adjusted for these individual factors. Further study of the impacts of social determinants of health and their association with clinical trial enrollment location may help optimize enrollment diversity for COVID-19 therapeutic trials and inform future clinical trial design cognizant of health equity.

Clinical trials or therapeutic delivery programs designed to reduce hospitalizations or death related to COVID-19 should consider incorporating AUEC enrollment settings into the enrollment and drug delivery design. Indeed, a trial with a comparable outcome to ACTIV-4B would require N = 557 participants per arm to have 80% power to detect a 50% reduction in the outcome with alpha = 0.05 if conducted using AUEC enrollment (outcome event risk 7.9% versus 3.95%) as compared to N = 6,689 participants per arm to detect a similar relative reduction (0.7% versus 0.35%) if conducted using list MC enrollment setting (Fig. 2).

### Limitations

The conclusions that may be drawn from ACTIV-4B are limited because the study was terminated early due to a paucity of composite outcome events and limits our ability to perform hierarchical analysis by enrollment location. The trial is unlikely to have enrolled a substantial number of patients with the Delta or omicron variants or full vaccination, and few patients who received monoclonal antibody infusions (e.g., bamlanivimab plus etesevimab), however this data is not available for our population and limits generalizability [14]. Identification of the primary composite outcome stratified by enrollment location was not planned a priori, and thus our observations are subject to biases of post-hoc analyses. For example, enrollment location was collected after the primary analyses and was available in 81% of trial participants, which may lead to residual confounding and precluded a direct causal relationship between the composite outcome and the location of enrollment. While we attempted to control for as many cofounders as practicable, we acknowledge that confounders likely existed that were outside the scope of our ability for control. Hospitalization was at the discretion of the individual site treating clinicians with inherent possibility of significant variability in admission criteria, and implementation of crisis standards that may have varied among the 52 enrolling sites. Symptom assessment was not obtained at time of enrollment so it is unknown whether symptoms may have been associated with the primary outcome. Finally, we did not capture the total number of AUEC encounters for either group; therefore, we cannot address whether multiple encounters impacted upon final admission rates.

## Conclusion

When compared to patients enrolled in an MC setting, patients with clinically stable COVID-19 presenting to an AUEC enrollment setting appear to be at increased risk of thrombosis complications, hospitalization for cardiopulmonary events or death, when adjusted for other risk factors. These findings have implications for the design of future outpatient therapeutic trials.

## Data Availability

Accession #: phs002710.v1. p1. **URL:** COVID-19 | BioData Catalyst (nih.gov). **Where to go to request access to the ACTIV-4B data URL:** dbGaP Study (nih.gov).

## Abbreviations

EHR: Electronic health record
MC: Minimal contact
AUEC: Acute unscheduled episode care
ED: Emergency departments
NHLBI: National Heart, Lung, and Blood Institute
MAB: Monoclonal Antibody
BMI: Body mass index
